# American Dental Association

**Published:** 1891-09

**Authors:** 


### Editorial.

### AMERICAN DENTAL ASSOCIATION.

The meeting of the American Dental Association, at Saratoga, was unquestionably one of the most valuable, in a scientific sense, that has been held for several years. The papers read were generally of a high order and indicated the growing strength of the profession in directions not heretofore thought attainable. Indeed it may be said, without fear of contradiction, that the whole tenor of the essays and discussions thereon were of a character not possible a decade ago. The days when it was thought necessary to spend the hours of a meeting of this kind in discussions on filling teeth or mechanical contrivances, important as these are, seem to have passed away, and the active mind concerns itself now with problems on therapeusis, surgery, and on those profound questions that relate to the origin of life.

The discussions as a whole were not as valuable as they might have been made. The excess of papers limited the time, and the unsatisfactory plan of reading these to an audience unfamiliar with the trend of the thoughts of the writers, left much to be desired in the remarks. Notwithstanding this drawback, the discussions on the comparative anatomy of the teeth and that on surgery held the audience as it could not have been held in past years. We were impressed with the remarks on the latter topic, as exhibiting much ability in the direction of oral surgery. They indicate that we are developing surgeons worthy the name. This was to have been expected, owing to the mechanical training of our profession, but it is none the less gratifying to know that this advance has been made to the degree demonstrated.

The meeting was largely attended and the feeling was apparent that the time had arrived for an advanced step, and this was taken in the appointment of a committee to revise the constitution. This movement was foreshadowed in the address of President Harlan. The old plan of "sections," originally adopted with so much enthusiasm, was regarded as a failure, and that the time had arrived for other modes of developing scientific work. The labor of this committee will be continued during the year with, we believe, satisfactory results, looking to the establishment of this Association on a more enduring foundation, and, let us hope, to a union of all the national societies.

There was a strong feeling in favor of making Washington, D. C., the home of the Association, and to meet there at least once in four years. There are some practical difficulties in the way in carrying this out, but we hope they may be overcome.

There was less friction than usual, and politics was at no time prominent. The long services of Dr. Walker received a proper recognition in his elevation to the presidency.

Saratoga destroys the social life of a convention, and this was the only unpleasant feature connected with the meeting. The conditions there prevent localization. The members scatter over the place, and have no opportunity for those reunions which made the meeting at Excelsior Springs, last year, one to be remembered.

